# Severe Mental Health Symptoms during COVID-19: A Comparison of the United Kingdom and Austria

**DOI:** 10.3390/healthcare9020191

**Published:** 2021-02-09

**Authors:** Sanja Budimir, Christoph Pieh, Rachel Dale, Thomas Probst

**Affiliations:** 1 Department for Psychotherapy and Biopsychosocial Health, Danube University Krems, 3500 Krems an der Donau, Austria; christoph.pieh@donau-uni.ac.at (C.P.); rachel.dale@donau-uni.ac.at (R.D.); thomas.probst@donau-uni.ac.at (T.P.); 2 Department of Work, Organization and Society, Ghent University, 9000 Gent, Belgium

**Keywords:** COVID-19 lockdown, mental health, depression, anxiety, insomnia, United Kingdom, Austria

## Abstract

This study evaluated severe psychological symptoms in the United Kingdom and Austria after four weeks of lockdown due to COVID-19. Two cross-sectional online surveys were performed with representative population samples according to age, gender, region, and education. Depressive symptoms were measured with the Patient Health Questionnaire (PHQ-9), anxiety symptoms with the Generalized Anxiety Disorder scale (GAD-7), and insomnia symptoms with the Insomnia Severity Index (ISI). The sample size was *N* = 1005 for Austria (52% women) and *N* = 1006 (54% women) for the UK. In total, 3.2% of the Austrian sample and 12.1% of the UK sample had severe depressive symptoms (PHQ-9 ≥ 20 points; χ^2^(1) = 57.24; *p* < 0.001), 6.0% in Austria vs. 18.9% in the UK had severe anxiety symptoms (GAD-7 ≥ 15 points; χ^2^(1) = 76.17; *p* < 0.001), and 2.2% in Austria and 7.3% in the UK had severe insomnia (ISI; ≥22 points; χ^2^(1) = 28.89; *p* < 0.001). The prevalence of severe depressive, anxiety or insomnia symptoms was around three times higher in the UK than in Austria.

## 1. Introduction

The Coronavirus disease 2019 (COVID-19) has spread quickly throughout the world [1]. In Europe alone, there are currently (26 January 2021) around 33 million confirmed COVID-19 cases and around 709,000 confirmed deaths [2]. The reported incidence rates across countries in Europe differed both during the first lockdowns in March/April of 2020 and to date (e.g., the UK has 1450 deaths per million vs. Austria (AT) with 822 deaths per million). According to the WHO/European COVID-19 official information (data as of 30 April 2020) the United Kingdom (UK) was the country with the most confirmed COVID-19 deaths (26,000) in Europe, while Austria (AT) was hit much less strongly by the COVID-19 pandemic, with 580 confirmed deaths at that time [2]. Taking the different population sizes of Austria (approximately nine million, with 65 deaths per million) and the UK (approximately 67 million, with 390 deaths per million) into account, during the first lockdown in March/April 2020, when this study was conducted, there were around six times more deaths due to COVID-19 in relation to the population in the UK than in Austria. 

As COVID-19 spreads easily between people who are in close contact [1], most governments have enacted restrictions to prevent the uncontrolled spread of the virus. The governmental restrictions due to COVID-19 started on different dates but were comparable in Austria and the UK. In Austria, measures against COVID-19 became obligatory on 16 March 2020. There were only five exceptions to the ban that allowed people to enter public places: activities to avert an immediate danger to life, limb, or property; professional activity (if working from home was not possible); errands to cover necessary basic needs; care and assistance for people in need of support; exercise outdoors (e.g., running, walking) alone and with pets/people living in the same household. A distance of at least 1 m to other people had to be ensured. In the UK, measures against COVID-19 became obligatory on 24 March 2020. Only the following exceptions allowed people to leave home: shopping for food and other necessities; exercising alone or with someone from the same household; attending to medical issues, including providing care to others; travelling to and from work. 

Although restrictions are effective in preventing the uncontrolled spread of COVID-19 [1], they might negatively affect mental health [3]. One of the adaptive reactions when facing a threat of infection is to conserve energy by withdrawing from activities and social interactions [4]. However, with the COVID-19 pandemic and the accompanying restrictions, social withdrawal is not imposed only upon those who have the virus, but to everyone. As active social support was found to be a predictor of better health and increased immunity [4], its reduction due to pandemic restrictions removes an additional protective factor that could be beneficial to certain individuals. Additionally, the COVID-19 pandemic resulted in the loss of different forms of resources, forcing individuals to cope with ongoing stressful circumstances. According to the Conservation of Resources (COR) theory [5], loss of resources (e.g., objects, personal characteristics, conditions or energies) presents a stressful event for individuals, requiring efficient coping mechanisms. Faced with stressful events, people engage in retaining, protecting and building resources, and they feel threatened and stressed if they encounter a loss [5]. Using thoughts and behaviours to deal with stressful situations, known as coping [6], can include attempts to change the relationship with the environment, change their interpretation of the environment [7], or avoidance [8]. The COVID-19 pandemic not only threatens a loss of built resources (through health and well-being threats, and lockdown restrictions that bring financial insecurity, relationship disruptions and a lack of social support, etc.), but also limits coping strategies due to disrupted life dynamics and lockdown restrictions. The role of resources and coping strategies in the stressful periods of a pandemic are crucial in helping us to understand how the pandemic can impact people’s emotional state, both in general populations and clinical samples. Coping with stressful life changes and a loss of resources can be especially challenging for initially more vulnerable groups (e.g., chronic arthritis patients are more likely to experience a lower body image, more intensive pain and lower COR [9]). Additionally, differences between individuals in the severity of their mental health problems could be expected depending on their differences in coping flexibility. When stressful life changes occur, some individuals are more likely to be focused on trying different coping strategies, while others may not be willing to engage themselves in the discovery of new strategies to cope with changed life circumstances and stressful events [10]. Coping flexibility and psychological adjustment are found to have a positive connection, especially in older people and people from countries with a low level of individualism [10]. 

The deteriorating effects of the COVID-19 pandemic on mental health became evident within the first few months of the pandemic [11,12,13,14,15,16,17,18,19,20]. An increased risk of anxiety, depression and sleep disorders were observed not only among healthcare workers working with people with COVID-19 [20], but also in the general population, especially in females, people with previous mental health problems [21] and chronic conditions [22], with indications of increased depression and anxiety compared to pre-COVID-19 levels [23]. A significant decline in mental well-being and an increase in depression were found across Asia, Africa and Europe, compared to measures prior to COVID-19 lockdowns [24]. 

The severity of the impact on Europe, reflected as the number of deaths, was the highest in the United Kingdom from the beginning of the pandemic, continuing for a period of ten months. Austria was among the first countries in Europe to introduce a lockdown relatively early, in March 2020, while still counting a low number of cases compared to the UK, which reacted later in terms of implementing its first lockdown measures. It is evident that many people’s mental health is deteriorating during the COVID-19 pandemic, and that this has to be taken into account when determining the restrictive and coping measures imposed upon general populations. This deteriorating trend might have even more damaging effects on individuals who already have fragile mental health. Therefore, we measured severe mental health symptoms, and compared them in two countries affected by COVID-19 to different degrees of severity, but with similar lockdown measures. In addition to the severe measures of depression, anxiety and insomnia, we also measured the perceived stress, well-being and quality of life of individuals with severe depression, anxiety and insomnia in Austria and the United Kingdom, after the countries had been in lockdown for approximately four weeks, in April 2020. Differences in mental health and the severity of depression, anxiety and insomnia between the two countries are expected as the severity of the impact of the COVID-19 pandemic (number of deaths) and the timing of the lockdown measures introduced differ between countries.

## 2. Materials and Methods

### 2.1. Study Design

Cross sectional online surveys targeting representative samples (according to age, gender, education, and region) were performed with Qualtrics^®^ [25] to measure mental health during the COVID-19 lockdown in the UK and AT. The surveys started after 4 weeks of quarantine in Austria as well as in the UK. COVID-19 lockdown became obligatory in Austria on 16 March 2020 (survey started on 10 April 2020) and in the UK on 24 March 2020 (survey started on 21 April 2020). Both surveys lasted 10 days. 

### 2.2. Study Sample

Representative samples according to age, gender, education, and region were recruited through a Qualtrics panel. Participants were contacted by the Qualtrics project team, who organized and coordinated data collection. We aimed for a representative sample size of at least 1000 participants in Austria (as well as in the UK), which resulted in an AT sample of 1005 and a UK sample of 1006 participants, representative of age, gender, education and region, for each country (for the sociodemographic characteristics of the samples, please see Supplementary Tables S2 and S3) The current paper focuses on severe cases of depression, anxiety, and insomnia. Table 1 and Table 2 show comparisons of socio-demographic variables between the UK and AT for severe cases of depression, anxiety and insomnia.

### 2.3. Measures

#### 2.3.1. Depressive Symptoms (PHQ-9)

Depressive symptoms were measured with the depression module of the Patient Health Questionnaire, the PHQ-9 [26], with 9 self-rating items on a four-point scale, from 0 to 3. Participants were asked if they have been affected by the problems listed over the last two weeks. A cut-off of greater than/equal to 20 points for severe depressive symptoms was used [27]. Cronbach’s alpha was α = 0.88 in the AT sample and α = 0.94 in the UK sample.

#### 2.3.2. Anxiety (GAD-7)

Anxiety symptoms were measured with the Generalized Anxiety Disorder scale (GAD-7) [28]. The GAD-7 is a validated instrument [29] that measures anxiety in the past two weeks with 7 self-rating items on a four-point scale, from 0 to 3. A cut-off point of at least 15 points was used to define severe anxiety symptom levels [28]. Cronbach’s alpha was α = 0.90 in the AT sample and α = 0.95 in the UK sample.

#### 2.3.3. Insomnia (ISI)

The Insomnia Severity Index (ISI) [30] is a self-reported 7-item measure of sleep quality and insomnia in the last two weeks. It is measured on a five-point scale (from 0 to 4). A cut-off score of at least 22 points was used to define severe insomnia symptoms [30]. Cronbach’s alpha was α = 0.84 in the AT sample and α = 0.91 in the UK sample.

#### 2.3.4. Quality of Life (WHOQOL-BREF)

The short version of the WHO Quality of Life questionnaire (WHOQOL-BREF) [31] provides a reliable, valid and brief assessment of quality of life for the period of the previous two weeks. The 26-item self-rating questionnaire measures physical health, psychological health, social relationships and environment during the past two weeks. WHOQOL-BREF has good to excellent psychometric properties of reliability and performed well in preliminary tests of validity [32]. We examined the psychological domain, for which the general norm is 70.6 (14.0) [33]. Cronbach’s alpha in the AT sample was α = 0.86 and in the UK sample it was α = 0.88.

#### 2.3.5. WHO-5 Well-Being

Well-being was measured with the WHO well-being questionnaire (WHO-5) [34]. It measures well-being within the past two weeks, with five self-rating items rated on a six-point Likert scale with a higher score indicating greater well-being. The WHO-5 has good psychometric properties [35,36]. The raw score ranges from 0 (absence of well-being) to 25 (maximal well-being). Because scales measuring health-related quality of life are conventionally translated to a percentage scale from 0 (absent) to 100 (maximal), it is recommended that the raw score be multiplied by 4 [36]. Cronbach’s alpha was α = 0.90 in the AT sample and α = 0.91 in the UK sample.

#### 2.3.6. Perceived Stress (PSS-10)

The subjective perception of stress levels was measured with a reliable and valid instrument that measures levels of stress over the period of the last month (the Perceived Stress Scale 10 (PSS-10)) [37]. Participants are asked to rate their stress level on a five-point scale (0–4) over ten items. Cronbach’s alpha was α = 0.89 in the AT sample and α = 0.88 in the UK sample.

### 2.4. Statistical Analysis

The data were analysed using SPSS version 24 (IBM Corp, Armonk, NY, USA) [38] and R version 4.0.2 [39]. Descriptive statistics were extracted to describe the demographic characteristics and mental health scales scores. Chi-squared tests were applied to investigate differences between the Austria and UK samples in terms of sociodemographic variables. In order to account for these differences between the samples, propensity score (PS) matching was run [39,40] to balance the differences between the two countries and further analyses were run both pre- and post-PS matching in order to assess the effects of the inherent differences between the two countries on the mental health outcome measures. The propensity score assigned to each participant represents the probability of belonging to one of the two groups, given a vector of observed covariates [41]. PS matching was conducted in R [40] using the MatchIt package and the subclass method, and age, gender, income, work status, education level and marital status were included as covariates. 

Adjusted residuals were used to interpret statistically significant differences between more than two groups, with values higher than 2 [42] indicating a statistically significant difference. Additionally, we included Fishers’ exact tests for higher accuracy in testing differences with small samples. T-tests for independent samples were calculated to compare the continuous mental health scale scores for severe cases of depression, anxiety and insomnia between Austria and the UK. Effect sizes were calculated (Hedges’ g), which can be interpreted as follows: small effect (g = 0.2–0.5), medium effect (g = 0.5–0.8), and large effect (g ≥ 0.8). In addition, chi-squared tests were applied to investigate differences between Austria and the UK in terms of depression, anxiety, and insomnia symptom severity categories (for cut-offs, see Section 2.3). *P*-values of less than 0.05 were considered statistically significant (2-sided tests).

### 2.5. Ethics Statement

The authors assert that all procedures contributing to this work comply with the ethical standards of the relevant national and institutional committees on human experimentation and with the Helsinki Declaration of 1975, as revised in 2008. All procedures involving human subjects/patients were approved by the Ethics Committee of the Danube University Krems, ethical number: EK GZ 26/2018-2021. Written informed consent was obtained from all subjects in electronic form. 

## 3. Results

The participants in both samples (AT and the UK), were recruited to reflect a representative ratio for gender, age, education and regions of the respective country (see [43,44] for both samples descriptions).

We calculated differences in terms of depression, anxiety and insomnia by comparing clinical cut-offs for severe cases and found significant differences between AT and the UK. Sample differences in sociodemographic variables were considered, and mental health differences were calculated for both before and after PS correction. In both cases, differences between the two samples were found to be statistically significant. As shown in Table 1, 3.1% of the Austrian sample and 12.1% of the UK sample scored above the PHQ-9 cut-off ≥ 20 for severe depressive symptoms (χ^2^(1) = 58.48; *p* < 0.001), indicating a four times higher prevalence of severe cases of depression in the UK. A comparison of severe cut-off for anxiety symptoms (above the GAD-7 cut-off ≥ 15), with 6.0% in Austria vs. 18.9% in the UK (χ^2^(1) = 77.05; *p* < 0.001), indicates a three times higher prevalence of severe anxiety cases in the UK. The same difference ratio was found for severe insomnia (above the ISI cut-off ≥ 22), with 2.2% in Austria and 7.3% in the UK (χ^2^(1) = 28.68; *p* < 0.001), indicating a three times higher prevalence of severe insomnia cases in the UK.

Based on the scores for the depression (PHQ-9), anxiety (GAD-7) and insomnia (ISI) measures within the representative samples for AT [43] and the UK [44], we selected participants with scores that indicated severe conditions in these measures. For each subsample with severe cases (depression, anxiety and insomnia), we calculated the differences in the sociodemographic characteristics between the two countries.

In the subsample with severe depressive cases (Table S1a), the only significant difference was found in education, with the UK subsample having fewer participants with an education level below high school (16.4%) compared to the AT subsample (41.9%).

In the subsample of severe anxiety cases (Table S1b), 35% of AT participants had an education level below high school, compared to 17.9% from the UK sample. Regarding work status, 10% of participants in the AT subsample had lost their jobs during the lockdown, compared to 26.8% in the UK subsample, while 31.7% of the AT subsample worked from home, compared to 14.7% in the UK subsample.

In the subsample of severe insomnia cases (Table S1c), 50% of AT participants had an education level below high school, compared to 19.2% in the UK subsample. In the AT subsample, 4.5% lost their job during the lockdown, compared to 24.7% in the UK subsample. Moreover, 22.7% of the AT subsample had a monthly income in the range of 3000–4000 EUR, compared to 2.7% in the UK subsample.

In order to account for these sociodemographic differences between the subsamples, but also not to ignore important cultural differences, we compared both pre- and post-PS matching in order to assess the effects of the inherent differences between the two countries on the mental health outcome measures, by taking into account age, gender, income, work status, education level and marital status differences between the samples. Although not statistically different between the two countries, age, gender, and marital status were included in the PS matching as they are considered highly relevant in mental health research [40]. 

We analysed differences in all measured mental health scales for the subsamples with severe cases of depression (Table 2a), anxiety (Table 2b) and insomnia (Table 2c).

In the subsample with severe depressive cases (Table 2a), the only significant differences in mean scores were found for: psychological health between the AT (M = 25.73, SD = 18.16) and UK subsamples (M = 36.00, SD = 23.29); well-being (AT (M = 4.29, SD = 3.18) compared to the UK subsample (M = 7.20, SD = 6.24)); and perceived stress (AT (M = 31.97, SD = 4.46) compared to the UK subsample (M = 27.21, SD = 5.79)). These results indicate that participants in the UK subsample with severe depressive cases have greater psychological health and well-being compared to the AT subsample, which has higher perceived stress. Differences in the mean values for depression, anxiety and insomnia scales were not significantly different between the severely depressive AT and UK subsamples.

We compared the same variables in the sample with severe anxiety cases (Table 2b). Significant differences in mean scores were found for: depression between the AT (M = 17.65, SD = 5.65) and UK subsamples (M = 19.54, SD = 23.29); anxiety (AT (M = 17.43, SD = 2.24) compared to the UK subsample (M = 18.26, SD = 2.19)); well-being (AT (M = 6.53, SD = 4.30) compared to the UK subsample (M = 7.67, SD = 5.71)); and perceived stress (AT (M = 29.17, SD = 5.51)) compared to the UK subsample (M = 26.51, SD = 5.72). These results indicate that participants in the UK subsample with severe anxiety cases have higher levels of depression and anxiety compared to the AT subsample. However, the UK severe anxiety subsample has a higher level of well-being compared to the AT subsample, while the AT subsample has a higher level of perceived stress than the UK sample.

There were no significant differences between the UK and AT severe insomnia subsamples on any of the measured mental health scales.

## 4. Discussion

The current study explored mental health in severe cases four weeks after the COVID-19 lockdowns in Austria and the UK. The COVID-19 pandemic, including the lockdowns, has had a major impact on mental health in both countries. However, mental health in the UK seems to be significantly worse compared to Austria. When comparing severe and non-severe cases of depression, anxiety and insomnia measures between the two countries, the UK has three times more severe cases for all three measures. The prevalence of severe depressive symptoms is around four times higher in the UK than in Austria, and the prevalence of severe anxiety and insomnia is around three times higher in the UK than in Austria in the current study. Unfortunately, there is a lack of research on the prevalence of severe depression, anxiety and insomnia in the pre-COVID-19 period, which would allow for a direct comparison with the prevalence of severe depressive, anxious and insomnia symptoms during COVID-19 lockdowns. Although there are no pre-COVID-19 epidemiological studies comparing severe levels of mental health symptoms, a comparison of the prevalence of severe depressive symptoms during the lockdown in the UK (12.1%) shows that their prevalence is higher than that of moderate depressive symptoms (UK: 7.4%, measured between 2013 and 2015) [45]. This indicates not only an increase in moderate depression, but in severe depression as well. In Austria, severe depressive symptoms (PHQ-9 ≥ 20 points) are now almost as prevalent (3.1) as mild to moderate depressive symptoms (PHQ-8 ≥ 10 points) were in 2014 (4.3%) [46]. Despite the lack of references for a direct comparison of severe symptoms pre-COVID-19, the prevalence of severe depression symptoms appears to be higher compared to pre-COVID-19 epidemiological studies of moderate depression. Moreover, a deterioration of mental health after the COVID-19 lockdown, compared to prior COVID-19 measures, was found in the form of increased depression and decreased well-being across Asia, Africa and Europe [24].

Additionally, differences between the two countries were expected in the subsamples with severe cases, based on the initial comparison of mental health measures in the general sample (Supplementary Tables S1–S3; also see [43,44]). The results for psychological life quality (Austria: 70 vs. the UK: 59; Hedges’ g: 0.56), well-being (WHO-5) (Austria: 15 vs. the UK: 13) and perceived stress (Austria: 16 vs. the UK: 18) indicated greater psychological quality, well-being and lower perceived stress in general in Austria compared to the UK during the lockdown. However, the same conclusion cannot be made when comparing AT and UK subsamples with severe depressive cases. UK participants with severe depression have increased psychological health and well-being and lower perceived stress compared to AT participants with severe depression. This result is unexpected if we take into account the mean values in the general sample for these mental health scales [43,44], which indicated the opposite—that UK participants have lower psychological health and well-being, as well as higher perceived stress. Additionally, when analysing subsamples with severe anxiety cases, although the UK subsample had higher depression and anxiety compared to the AT subsample, the UK severe anxiety subsample showed greater well-being compared to the AT subsample. Similar to the AT severe depressive subsample, the AT severe anxiety subsample also had higher perceived stress compared to the UK subsample. A possible explanation of the lower well-being and psychological health within AT, in both the severe anxiety and severe depressive subsamples, could be the higher perception of stress in the AT subsamples, as a negative correlation of perceived stress and well-being has been found in other studies [47,48]. Comparisons of subsamples with severe insomnia did not reveal any significant differences between UK and AT subsamples on any of the measured mental health scales.

Some explanations can be offered for the differences in the prevalence rates of severe cases of depression, anxiety and insomnia between Austria and the UK in the general sample. First, and most obviously, is the varied severity of the pandemic in the two countries. The UK was hit much harder by the COVID-19 pandemic than Austria. These mental health indicators were measured during the lockdown in both countries and, in that period, there was a significant difference between the UK (more than 28,000) and Austria (580) in the number of confirmed deaths due to COVID [49]. Considering the different population sizes of Austria (approximately nine million) and the UK (approximately 67 million), in the measured period, there were six times more deaths due to COVID-19 in relation to the population in the UK than in Austria. Secondly, we performed the survey after four weeks of lockdown in Austria and the UK. At that time, in Austria, the spread of the COVID-19 pandemic had already flattened, whereas, in the UK, it was still increasing. Thirdly, although we addressed sociodemographic differences by calculating PS, and testing mental health differences before and after PS matching, job situation, income and education are likely to impact one’s mental health situation, as shown by several previous studies [50,51,52].

When interpreting the results, the following limitations should be considered. This cross-sectional study allows no causal conclusions to be made. Two measurement points (before vs. during COVID-19 lockdown) would have been more adequate to study changes in mental health. We can compare our results only to the available prevalence measures from the pre-COVID-19 period. Moreover, the online survey was based only on self-rating tests. Although valid and widely used, people are often biased when they report on their own experiences [53]. A clinician’s assessment, which is necessary to make statements about psychiatric disorders, such as the Structured Clinical Interview, was missing, which limits the interpretation of the results. Furthermore, the current results were compared to previous studies, which were (in general) conducted some years earlier, and in other countries. It remains unclear which effects are explained by the current COVID-19 situation. A major limitation of this study is that there were no measures applied to assess the degree to which participants were individually affected by the lockdown restrictions. Therefore, we cannot answer the question of the associations between mental health and individual lockdown-related problems. Additionally, individual differences in regard to chronic conditions, coping flexibly and the availability of resources [5,9,10] in stressful situations should be taken into account in future studies. We only used questionnaires that are available and validated in English and German; however, a general country effect cannot be excluded. Furthermore, due to Brexit, which recently came into effect, the UK is undergoing a process of change. It is unclear whether the mental health scores tested were comparable between Austria and the UK before COVID-19. 

Our results imply that there is a need for resources [5] and coping strategies [6,7] to be implemented in order to help people handle their mental health problems. In particular, for the COVID-19 pandemic suggestions include managing expectations, proactive management of one’s stress threshold, knowing one’s red flags, maintaining a routine, showing compassion, and making connections with others, as well as practising mindfulness [54]. Additional attention should be given to severe cases, as there is a trend of mental health deterioration caused by lockdown extensions [55], which can make borderline cases vulnerable to the development of more severe symptoms.

## 5. Conclusions

There is an evident difference in mental health problems during lockdown due to the COVID-19 pandemic between Austria and the UK. The more a country is affected by a pandemic, the more mental health seems to be impaired. The UK has been hit five times harder than Austria, according to the death rates due to COVID-19 in relation to the total population (WHO/Europe COVID-19 website), and three to four times harder according to severe mental health symptoms. However, when comparing mental health within subsamples with severe cases, the severe depressive subsample in the UK had greater psychological health and well-being, while the AT subsample had higher perceived stress levels. The severe anxiety UK subsample had higher levels of depression and anxiety, but also greater well-being compared to the AT subsample, which had higher perceived stress, while severe insomnia subsamples did not reveal between-countries differences. To counteract the increased burden of mental health problems, timely mental health care should urgently be offered in both the UK and Austria [56]. 

## Figures and Tables

**Table 1:** Differences in severe symptoms of depression, anxiety, and insomnia between Austria and the UK after 4 weeks of COVID-19 lockdown.

Scale	Sample		Statistic	
	Austria *N* = 1005	UK *N* = 1006	Before PS Correction	After PS Correction
Measures f (%)				
PHQ-9				
Not severe (<20 points)	974 (96.9)	884 (87.9)	χ^2^(1) = 58.48; *p* < 0.001	χ^2^(1) = 48.52; *p* < 0.001
Severe (≥20 points)	31 (3.1)	122 (12.1)		
GAD-7				
Not severe (<15 points)	945 (94.0)	816 (81.1)	χ^2^(1) = 77.05; *p* < 0.001	χ^2^(1) = 63.52; *p* < 0.001
Severe (≥15 points)	60 (6.0)	190 (18.9)		
ISI				
Not severe (<22 points)	983 (97.8)	933 (92.7)	χ^2^(1) = 28.68; *p* < 0.001	χ^2^(1) = 24.37; *p* < 0.001
Severe (≥22 points)	22 (2.2)	73 (7.3)		

f: frequencies; %: percent; *p*: *p*-values (2-tailed); ISI: Insomnia Severity Index, GAD-7: Generalized Anxiety Disorder 7 scale; PHQ-9: Patient Health Questionnaire 9 scale; χ^2^: chi-square, PS: Propensity Score.

**Table 2:** Comparisons between continuous mental health scores of Austrian and UK samples in those with severe PHQ9 depressive symptoms. **b.** Comparisons between continuous mental health scores of Austrian and UK samples in those with severe GAD-7 anxiety symptoms. **c.** Comparisons between continuous mental health scores of Austrian and UK samples in those with severe ISI insomnia symptoms.

**a**				
**Scale**	**Sample**		**Statistic**	
	**Austria** ***N* = 31**	**UK** ***N* = 122**	**Before** **PS Correction**	**After** **PS Correction**
Depression (PHQ-9) M (SD)	22.71 (2.21)	23.27 (2.52)	t(51.71) = −1.23; *p* > 0.05; g = 0.23	t(150) = −1.15; *p* > 0.05; g = 0.23
Anxiety (GAD-7) M (SD)	17.68 (3.10)	17.70 (3.68)	t(53.60) = −0.04; *p* > 0.05; g = 0.01	t(150) = −0.11; *p* > 0.05; g = 0.01
Insomnia (ISI) M (SD)	17.71 (5.92)	18.05 (6.15)	t(47.81) = −0.28; *p* > 0.05; g = 0.06	t(150) = −0.32; *p* > 0.05; g = 0.06
Psychological Health (WHOQOL BREF) M (SD)	25.73 (18.16)	36.00 (23.29)	t(57.81) = −2.65; *p* = 0.01; g = 0.99	t(150) = −2.6; *p* < 0.01; g = 0.46
Well-Being (WHO-5) M (SD)	4.29 (3.18)	7.20 (6.24)	t(95.12) = −3.63; *p* < 0.001; g = 0.50	t(150) = −2.78; *p* < 0.01; g = 0.50
Stress (PSS-10) M (SD)	31.97 (4.46)	27.21 (5.79)	t(58.55) = −4.97; *p* < 0.001; g = 0.85	t(150) = 4.54; *p* < 0.0001; g = −0.85
**b**				
**Scale**	**Sample**		**Statistic**	
	**Austria** ***N* = 60**	**UK** ***N* = 190**	**Before** **PS correction**	**After** **PS correction**
Depression (PHQ-9) M (SD)	17.65 (5.65)	19.54 (5.45)	t(96.07) = −2.27; *p* = 0.03; g = 0.34	t(247) = −2.33; *p* < 0.05; g = 0.34
Anxiety (GAD-7) M (SD)	17.43 (2.24)	18.26 (2.19)	t(97.04) = −2.50; *p* = 0.01; g = 0.37	t(247) = −2.5; *p* < 0.01; g = 0.37
Insomnia (ISI) M (SD)	16.08 (6.51)	16.71 (6.54)	t(99.38) = −6.50; *p* > 0.05; g = 0.09	t(247) = −6.27; *p* > 0.05; g = 0.09
Psychological Health (WHOQOL BREF) M (SD)	38.04 (19.34)	37.17 (20.37)	t(103.63) = 0.30; *p* > 0.05; g = 0.04	t(247) = 2.63; *p* > 0.05; g = 0.04
Well-Being (WHO-5) M (SD)	6.53 (4.30)	7.67 (5.71)	t(130.29) = −1.65; *p* < 0.01; g = 0.21	t(247) = 2.94; *p* < 0.05; g = 0.21
Stress (PSS-10) M (SD)	29.17 (5.51)	26.51 (5.72)	t(102.37)= 3.23; *p* < 0.01; g = 0.47	t(247) = 3.48; *p* < 0.001; g = −0.47
**c**				
**Scale**	**Sample**		**Statistic**	
	**Austria** ***N* = 22**	**UK** ***N* = 73**	**Before** **PS correction**	**After** **PS correction**
Depression (PHQ-9) M (SD)	16.91 (7.14)	18.55 (7.21)	t(34.95) = −0.94; *p* > 0.05; g = 0.23	t(92) = −1.13; *p* > 0.05; g = 0.23
Anxiety (GAD-7) M (SD)	14.73 (4.89)	15.55 (6.03)	t(42.07) = −0.65; *p* > 0.05; g = 0.14	t(92) = −0.72; *p* > 0.05; g = 0.14
Insomnia (ISI) M (SD)	23.95 (1.84)	24.42 (2.09)	t(38.79) = −1.02; *p* > 0.05; g = 0.23	t(92) = −1.08; *p* > 0.05; g = 0.23
Psychological Health (WHOQOL BREF) M (SD)	46.14 (23.95)	39.67 (21.93)	t(32.35) = 1.13; *p* > 0.05; g = 0.29	t(92) = 0.57; *p* > 0.05; g = −0.29
Well-Being (WHO-5) M (SD)	7.27 (5.25)	7.71 (6.17)	t(40.14) = −0.33; *p* > 0.05; g = 0.07	t(92) = −0.78; *p* > 0.05; g = 0.07
Stress (PSS-10) M (SD)	27.55 (7.35)	25.52 (6.27)	t(30.80) = 1.17; *p* > 0.05; g = 0.31	t(92) = −1.93; *p* > 0.05; g = −0.31

M: mean score; SD: standard deviation, *p*: *p*-values (2-tailed); *T*: *T*-test; g: Hedges’ g; ISI: Insomnia Severity Index, GAD-7: Generalized Anxiety Disorder 7 scale; PHQ-9: Patient Health Questionnaire 9 scale; PSS-10: Perceived Stress Scale 10; WHO-5: WHO well-being questionnaire; WHOQOL-BREF: short version of the WHO Quality of Life questionnaire, PS: Propensity Score.

## Data Availability

The data presented in this study are available in the Supplementary Materials.

## Supplementary Materials

The following are available online at https://www.mdpi.com/2227-9032/9/2/191/s1, Table S1a. Comparisons between the Austrian and UK severe depressive cases by sociodemographic variables (gender, age, education and region). Table S1b. Comparisons between the Austrian and UK severe anxiety cases by sociodemographic variables (gender, age, education and region). Table S1c. Comparisons between the Austrian and UK severe insomnia cases by sociodemographic variables (gender, age, education and region). Table S2. Comparisons between the Austrian and UK depressive severe cases by sociodemographic variables (gender, age, education and region). Table S3. Comparisons of work, income and marital status between the Austrian and UK samples. Table S4. Comparisons of continuous mental health scale scores between the Austrian and UK samples.
